# Impact of Fenofibrate on Type 2 Diabetes Patients with Features of the Metabolic Syndrome: Subgroup Analysis From FIELD

**DOI:** 10.2174/157340310791162686

**Published:** 2010-05

**Authors:** Michel P. Hermans

**Affiliations:** Cliniques Universitaires St-Luc, Service d’Endocrinologie et Nutrition, Brussels, Belgium

**Keywords:** Metabolic syndrome, cardiovascular risk, PPARα agonists, fenofibrate, type 2 diabetes.

## Abstract

Given evidence of increasing prevalence in developed and developing countries, as a result of obesity trends and sedentary lifestyles, the metabolic syndrome represents an increasing burden on healthcare systems. Management guidelines for dyslipidaemia have primarily focused on LDL-C reduction; however, this approach fails to sufficiently address other lipid abnormalities associated with the metabolic syndrome. Atherogenic dyslipidaemia (characterized by elevated triglycerides and low HDL-C) is strongly associated with insulin-resistant states, such as type 2 diabetes and the metabolic syndrome, and is also a common finding among patients receiving treatment for dyslipidaemia. Intervening against atherogenic dyslipidaemia may address a substantial modifiable fraction of residual cardiovascular risk that remains after treatment with a statin. Recent findings from the Fenofibrate Intervention and Event Lowering in Diabetes (FIELD) study support this view. Fenofibrate treatment was shown to be especially effective in treating marked atherogenic dyslipidaemia, with a significant 27% relative risk reduction for cardiovascular events (P=0.0005, vs. 11%, P=0.035 for all patients) relative to placebo. These data, together with the earlier demonstration of significant microvascular benefits associated with this treatment, suggest a role for fenofibrate, in addition to statin therapy and lifestyle intervention, for reducing global vascular risk in type 2 diabetes patients and for impacting atherogenic dyslipidaemia associated with the metabolic syndrome.

## INTRODUCTION

While mortality rates from cardiovascular disease have declined in recent decades, global trends including increasing prevalence of type 2 diabetes and the continuing tendency towards the adoption of sedentary lifestyles and high-energy diets in developing countries, threaten to slow or even reverse this progress. There is no doubt that aggressive multifactorial intervention to address cardiometabolic risk factors can have a major impact on the risk of adverse cardiovascular outcomes, and continuing research will refine our ideas on how, when and to whom to apply such interventions. This is especially relevant for individuals with type 2 diabetes and/or the metabolic syndrome. Recent analyses from the large Fenofibrate Intervention and Event Lowering in Diabetes (FIELD) study have shed new light on the management of cardiovascular risk in this population. This review examines these new data in the light of the management of cardiovascular risk in patients with type 2 diabetes and features of the metabolic syndrome.

## CURRENT FOCUS OF LIPID-MODIFYING THERAPY

Current guidelines for the management of the lipid profile continue to strongly focus on the control of cholesterol associated with the major circulating atherogenic lipoprotein (i.e. apolipoprotein B_100_ [ApoB]-containing lipoproteins, mostly low-density lipoproteins [LDL] and/or of total cholesterol). The US National Cholesterol Education Program/Adult Treatment Panel III (NCEP/ATP III) recommends that “LDL-cholesterol (LDL-C) be the primary target of therapy [1].” Goals for LDL-C are provided for different risk categories: <100 mg/dL (2.6 mmol/L) for patients with coronary heart disease (CHD) or CHD equivalents (such as type 2 diabetes), <130 mg/dL (3.4 mmol/L) for patients with two or more standard cardiovascular risk factors, and <160 mg/dL (4.1 mmol/L) for patients with no more than one cardiovascular risk factor. The guidelines from the European Society of Cardiology (ESC) recommend management of cholesterol within the broader framework of global cardiovascular risk, including overweight, obesity, hypertension and smoking, with cholesterol goals <5 mmol/L (about 190 mg/dL) for total cholesterol and <3 mmol/L (about 115 mg/dL) for LDL-C in low-risk patients [2]. Again, there are more stringent goals for patients in higher cardiovascular risk categories of <4-4.5 mmol/L (about 155–175 mg/dL) and 2-2.5 mmol/L (about 80-100 mg/dL) where feasible.

Non-HDL-C (the combined cholesterol load from very low-density lipoproteins [VLDL], intermediate-density lipoproteins [IDL], LDL and other remnants) was proposed as secondary target for therapy beyond LDL-C in the NCEP/ATPIII guidelines for people with triglycerides ≥2.3 mmol/L (≥200 mg/dL) [1]. One rationale was that as triglycerides rise, LDL-C estimation using Friedewald’s formula tends to underestimate the true levels of LDL-C. Another is that non-HDL-C provides additional information on the level of all atherogenic lipoproteins, including those of intermediate size generated during ongoing triglycerides removal (such as IDL) and other ApoB-containing remnants, and correlates closely with ApoB levels. In addition, these guidelines provide some support for intervention to correct atherogenic dyslipidaemia (low HDL-C and/or high triglycerides), with primary emphasis on lifestyle intervention. HDL-C <1.03 mmol/L (40 mg/dL) and <1.29 (50 mg/dL) is recognized as ‘too low’ for men and women, respectively, although no specific goal value for raising this parameter was identified as target for therapy. Similarly, European guidelines for cardiovascular risk reduction do not propose goal values for HDL-C or triglycerides [2]. 

In practice, control of LDL-C usually means intervention with a statin (occasionally combined with ezetimibe) where lifestyle interventions (diet and exercise) are insufficiently effective. There is no doubt that this strategy has delivered substantial reductions in the risk for cardiovascular events in at-risk populations, both in primary and secondary cardiovascular prevention. Large prospective studies have demonstrated relative reductions in risk of cardiovascular events of 20–50% depending on the baseline level of risk [3]. 

## ATHEROGENIC DYSLIPIDAEMIA, METABOLIC SYNDROME AND CARDIOVASCULAR RISK

### Diagnosing the Metabolic Syndrome

The metabolic syndrome represents both an important source as well as an estimate of global cardiovascular risk in addition to hypercholesterolaemia and other standard risk factors, such as smoking and familial cardiovascular history. The condition describes a clustering of cardiovascular risk factors in patients with insulin-resistant states, including obesity, hypertension, coronary heart disease and/or type 2 diabetes. The most commonly-used diagnostic criteria for the metabolic syndrome have been proposed jointly by various scientific bodies, such as the NCEP/ATPIII, the American Heart Association (AHA/NHLBI) [1, 4], and the International Diabetes Federation (IDF) [5]. Each presents a set of five individual criteria based on levels of (or specific treatment for) waist circumference, HDL-C, triglycerides, fasting glucose and blood pressure, and requires the presence of three or more of these five easily-obtained anthropometric or biochemical cardiovascular risk factors to support a diagnosis of the metabolic syndrome.

The main difference between these definitions in terms of practical diagnosis is that meeting any three criteria is sufficient to support the diagnosis of metabolic syndrome according to the NCEP/ATPIII or the AHA/NHLBI, while the IDF require abdominal obesity (high waist circumference) as *sine qua non* criterion plus two or more other criteria. In addition, the IDF provide lower and ethnic-specific cut-offs for waist circumference relative to the NCEP/ATPIII. Other differences include lower cut-off values for fasting glucose (although a threshold of 100 mg/dL was subsequently endorsed by other definitions), and the inclusion of treatment for any specific abnormality in addition to absolute levels of metabolic parameters, in the IDF criteria.

### High Prevalence of Metabolic Syndrome in Type 2 Diabetes Patients

The prevalence of the metabolic syndrome in non-diabetic patients in western countries is in the region of 25–35%, depending on the diagnostic criteria used [6-8]. However, prevalence appears to be considerably higher in patients with type 2 diabetes. For example, about 80% of type 2 diabetes patients from Finland or Sweden in the Botnia cohort had metabolic syndrome [6]. Additionally, high prevalence rates of NCEP/ATPIII metabolic syndrome have been demonstrated among the populations of major clinical trials in exclusively type 2 diabetes populations, such as the United Kingdom Prospective Diabetes Study (UKPDS) (61%) [9], and the FIELD study (>80%), the latter population being largely without cardiovascular disease at baseline [10].

These observations are consistent with the underlying pathology of type 2 diabetes. Patients with type 2 diabetes meet the diagnostic criterion relating to hyperglycaemia, by definition. In addition, both hypertension and an atherogenic dyslipidaemia phenotype (characterized by low HDL-C, elevated triglycerides and an increase in small, dense LDL), are associated strongly with insulin resistance and compensatory hyperinsulinaemia. These abnormalities are the hallmark of many insulin-resistant states, such as the metabolic syndrome and the common form of type 2 diabetes [11, 12]. A survey conducted in >8000 patients receiving treatment for dyslipidaemia, (mostly with a statin) in 11 European countries confirmed a higher prevalence of low HDL-C and/or hypertriglyceridaemia in patients with type 2 diabetes, relative to patients without diabetes (Table **1**) [13, 14]. 

National Cholesterol Education Program/Adult Treatment Panel III cut-off values used to determine low or high lipid parameters. Type 2 diabetes was present in 3866 patients and absent in 4436 patients. HDL-C: high-density lipoprotein cholesterol; TG: triglycerides. Data from Bruckert *et al*. [13,14].

### Metabolic Syndrome and Adverse Cardiovascular Outcomes

**Fig. (1):**
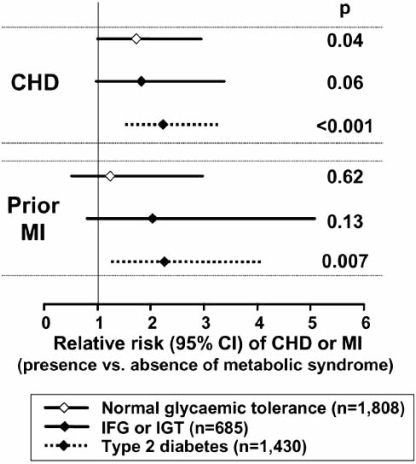
Impact of the metabolic syndrome on the risk of cardiovascular disease according to glycaemic status in the Botnia Study. Coronary heart disease (CHD) was defined as use of nitroglycerine or prior myocardial infarction (MI). Relative risks were adjusted for age and gender. IFG: impaired fasting glucose; IGT: impaired glucose tolerance. Drawn from data presented by Isomaa *et al.* [6].

A number of studies have associated the metabolic syndrome significantly and independently with an increased risk for type 2 diabetes or cardiovascular disease [15-18]. Importantly, the presence of the metabolic syndrome accentuates cardiovascular risk associated with type 2 diabetes, seemingly above the summative risk afforded by its discrete components. An analysis from the Botnia cohort illustrates this point. Within a cohort of 4483 subjects aged 35–70 years, the presence of the metabolic syndrome (diagnosed using alternative criteria from the World Health Organisation) increased the risk of pre-existing coronary heart disease in patients with normal glucose homeostasis or with pre-diabetic states (impaired fasting glucose and/or impaired glucose tolerance), or a clinical diagnosis of type 2 diabetes (Fig. **1**). The presence of the metabolic syndrome also increased the risk of stroke by about 2–fold in the overall population (P<0.001), although increases in relative risk associated with the metabolic syndrome did not achieve statistical significance after stratification of the population for glycaemic status.

Longitudinal data confirm the strong association between the metabolic syndrome and adverse cardiovascular outcomes in patients with type 2 diabetes. A *post-hoc* analysis from the UKPDS studied the incidence of micro- and macrovascular complications with respect to baseline metabolic syndrome in a subset of 4542 newly-diagnosed type 2 diabetes patients over an average follow-up duration of 10 years [9]. The risk of macrovascular complications was markedly and significantly higher in patients with metabolic syndrome, with increases in the risk of cardiovascular disease or myocardial infarction of about 30% and an increase in the risk of stroke of about 70% in patients fulfilling NCCEP/ATPIII criteria. Although the risk of microvascular complications was not captured by metabolic syndrome identification in the UKPDS, other studies, for example the Metascreen study [19], have reported an increased risk of macrovascular and microvascular complications in type 2 diabetic patients with metabolic syndrome defined according to AHA/NHLBI or IDF criteria.

**Fig. (2):**
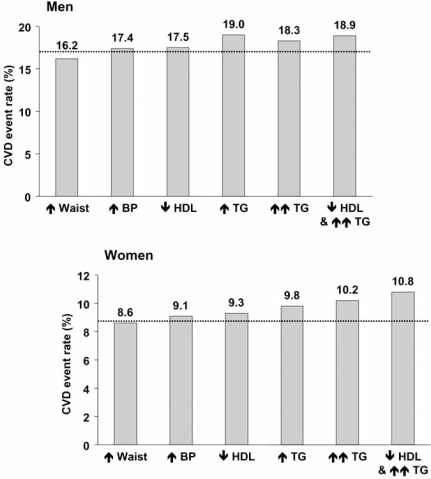
Cardiovascular disease (CVD) event rates in patients with features of the metabolic syndrome in the FIELD study. Dotted lines represent CVD event rates in patients fulfilling the criteria for National Cholesterol Education Program/Adult Treatment Panel III metabolic syndrome. Individual criteria were as follows: **↑** waist: waist circumference >102 cm for men or >88 cm for women; **↑** BP ≥135/≥85 mmHg; **↓** HDL: HDL-C <1.03 mmol/L for men or <1.29 mmol/L for women; **↑** TG: triglycerides ≥1.7 mmol/L; **↑↑** TG: triglycerides >2.3 mmol/L. Drawn from data presented by Scott *et al.* [10].

In the FIELD study, features of atherogenic dyslipidaemia (hypertriglyceridaemia and/or low HDL-C) were more strongly associated with increased cardiovascular event rates than the two other, non-glycaemic diagnostic criteria for the metabolic syndrome (Fig. **2**). Incident cardiovascular disease rate increased in line with the number of non-glycaemic metabolic syndrome criteria in addition to type 2 diabetes in this study. Relatively little interaction was observed between high blood pressure and the metabolic syndrome, as cardiovascular event rates were only about 10% higher in hypertensive patients with *vs.* without the metabolic syndrome. Similarly, the presence of the metabolic syndrome increased cardiovascular event rates by about 30% relative to patients without the metabolic syndrome in patients with abdominal obesity. However, the greatest impact of the metabolic syndrome on cardiovascular prognosis was observed in patients with marked hypertriglyceridaemia (≥2.3 mmol/L or 200 mg/dL) or low HDL-C, where the presence of the metabolic syndrome increased cardiovascular event rates by about 2.8-fold and by about 2-fold, respectively.

Whether the metabolic syndrome is itself a driver of excess cardiovascular risk, or whether the individual risk factors included in its diagnostic criteria account for all of the excess adverse cardiovascular outcomes in these patients is contentious [20, 21]. It is important to note here that the diagnostic criteria of the metabolic syndrome represent only a few of the metabolic abnormalities associated with it. A range of other cardiovascular risk factors also tends to cluster in patients with the metabolic syndrome, such as a prothrombotic state, oxidative stress, endothelial dysfunction, low-grade systemic inflammation and/or albuminuria. These are all likely to contribute to (or reflect) to a greater or lesser extent the development (or the burden) of premature atherosclerotic cardiovascular disease [22].

## REDUCING CARDIOVASCULAR RISK IN TYPE 2 DIABETES PATIENTS WITH METABOLIC SYNDROME

### Pathophysiologic Considerations

The data summarized above suggest that the presence of the metabolic syndrome is likely to be useful in identifying type 2 diabetes patients at markedly increased cardiovascular risk who would benefit from intensive intervention [23]. Identifying a metabolic syndrome phenotype may be used either as a dichotomic state (presence or absence), whereas score ranking within metabolic syndrome patients may even represent stepwise rise in cardiovascular risk (from 3/5 to 5/5). The data from the FIELD study, in particular, highlight atherogenic dyslipidaemia as an important source of modifiable cardiovascular risk in this population. Thus, treatments which target atherogenic dyslipidaemia and other risk factors associated with type 2 diabetes and the metabolic syndrome represent a rational approach to the management of this population. Peroxisome proliferator activator receptor-α (PPARα), a nuclear receptor with important transcriptional regulator activity on lipid metabolism, energy balance and inflammation, has emerged as a logical target for therapeutic intervention in metabolic syndrome and type 2 diabetes [24, 25]. As pharmacological agents, fibric acid derivatives exert their therapeutic action by activating PPARα. Fenofibrate, a member of this therapeutic class, is a PPARα ligand which has been shown to increase levels of HDL-C by 10–15% and to reduce triglycerides by up to 50%, dependent on baseline levels [26-28]. In addition, fenofibrate induces a shift in the subclass distribution of LDL from small, dense LDL to larger particles, usually associated with lesser atherogenic profiles [29, 30].

Fig. (**3**) shows the effects of fenofibrate, simvastatin and both agents in combination, on these parameters in a 12-week, randomized trial in patients with type 2 diabetes and mixed dyslipidaemia [28, 30]. Fenofibrate was more effective than simvastatin in increasing HDL-C and decreasing triglycerides, and simvastatin was more effective in reducing LDL-C, as would be expected from their mode of action. However, fenofibrate, but not simvastatin, significantly decreased the proportion of LDL in the small, dense (pattern B) subclass, and increased the proportion of larger, more buoyant LDL. Overall, the combination treatment resulted in the least atherogenic lipid profile. These and other studies have also shown that treatment with fenofibrate reduced a series of markers of chronic low-grade systemic inflammation, which have been implicated in the pathogenesis of atherosclerosis or thought of as reflecting its burden [25, 26, 31, 32]. Thus, fenofibrate modulates both key factors and markers of cardiovascular risk associated with the metabolic syndrome that are not adequately addressed by statins.

## EXPERIENCE FROM THE FIELD STUDY

### Overview of FIELD

In the FIELD study, 9795 patients with type 2 diabetes were randomized to receive treatment with fenofibrate or placebo for an average of 5 years [33, 34]. The study population as a whole was at relatively low cardiovascular risk; patients with an indication for treatment with a statin at baseline were ineligible for the trial, and only 22% had pre-existing cardiovascular disease. Although a reduction of 11% vs. placebo in the primary composite endpoint (nonfatal myocardial infarction or coronary heart disease death) did not achieve statistical significance (hazard ratio [HR] 0.89 [95% CI 0.75 to 0.15], P=0.16), randomization to fenofibrate was associated with significant reductions in the risk of nonfatal myocardial infarction (-24%; HR 0.76 [95% CI 0.62 to 0.94], p=0.01), any cardiovascular disease event (-11%; HR 0.89 [95% CI 0.80 to 0.99], P=0.035), coronary revascularization (-21%; HR 0.79 [95% CI 0.68 to 0.93], P=0.003) and any revascularization (-20%; HR 0·80 [95% CI 0.70 to 0.92], p=0.001). A marked reduction in microvascular complications was also observed in the fenofibrate group, including reductions relative to placebo in risk of retinopathy (especially in patients without prior retinopathy), lower-limb non-traumatic amputation, or progression of nephropathy markers [35, 36]. Fenofibrate was well tolerated, with an incidence of side-effects similar to placebo. In particular there was no excess of musculoskeletal serious adverse events with fenofibrate (15% in either group) and only four cases of rhabdomyolysis occurred (one in a patient on placebo and three in patients on fenofibrate, none of whom were receiving a statin).

### Patients With Features of the Metabolic Syndrome

The latest analysis from the FIELD study concerns the effects of fenofibrate in patients with cardiovascular risk factors featuring the metabolic syndrome [10]. As expected in a type 2 diabetes population, the proportions of patients who fulfilled diagnostic criteria for NCEP/ATPIII metabolic syndrome were high and similar in the placebo (84%) and fenofibrate groups (83%). More women (91%) than men (79%) had the metabolic syndrome at baseline. All patients met the diagnostic criterion relating to hyperglycaemia, as this was an exclusively type 2 diabetes population. More women than men were abdominally obese (83% and 59%, respectively) or had low HDL-C (67% and 55%, respectively); similar proportions of women and men had hypertriglyceridaemia (55% and 50%, respectively) or high blood pressure (86% and 82%, respectively).

The effects of fenofibrate on the risk of incident cardiovascular events in patients stratified for the presence of various features of the metabolic syndrome are shown in Fig. (**4**). A risk reduction of 11% for fenofibrate vs. placebo in patients with the metabolic syndrome was similar in magnitude to the effect of fenofibrate in the overall population, but marginally failed to reach statistical significance (P=0.052). With regard to individual metabolic syndrome criteria, significant adjusted risk reductions were observed in patients with raised blood pressure and low HDL-C (–12% and -14%, respectively). However, the largest risk reductions occurred in patients with coexisting marked hypertriglyceridaemia and low HDL-C, representing the most comorbid combination in atherogenic dyslipidaemia (-27%, P=0.005). The magnitude of this reduction was in line with the risk reduction observed in landmark statin trials performed in nondiabetic and diabetic cohorts.

## CONCLUSIONS

Atherogenic dyslipidaemia is commonly observed among patients under treatment for dyslipidaemia and is an important effector of cardiovascular risk. Recent findings from the FIELD study indicate that not only are type 2 diabetes patients with atherogenic dyslipidaemia at greatest risk for cardiovascular events, but that fenofibrate was most effective in this population. These data, together with the earlier demonstration of significant microvascular benefits associated with this treatment suggest a role for fenofibrate, in addition to statin therapy and lifestyle intervention, for reducing this residual vascular risk in type 2 diabetes patients with atherogenic dyslipidaemia.

## Figures and Tables

**Fig. (3):**
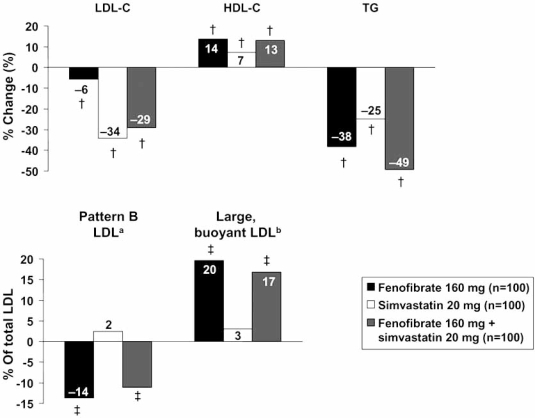
Effects of fenofibrate on lipid parameters and LDL subclass profile in a randomized trial in patients with mixed hyperlipidaemia. ^a^LDL_3_ and LDL_4_ cholesterol; ^b^LDL_1_ and LDL_2_ cholesterol. †P<0.001; ‡P<0.0001 vs. baseline. HDL-C: high-density lipoprotein cholesterol; LDL-C: low-density lipoprotein cholesterol, TG: triglycerides. Drawn from data presented by Muhlestein *et al.* [28] and May *et al.* [30].

**Fig. (4):**
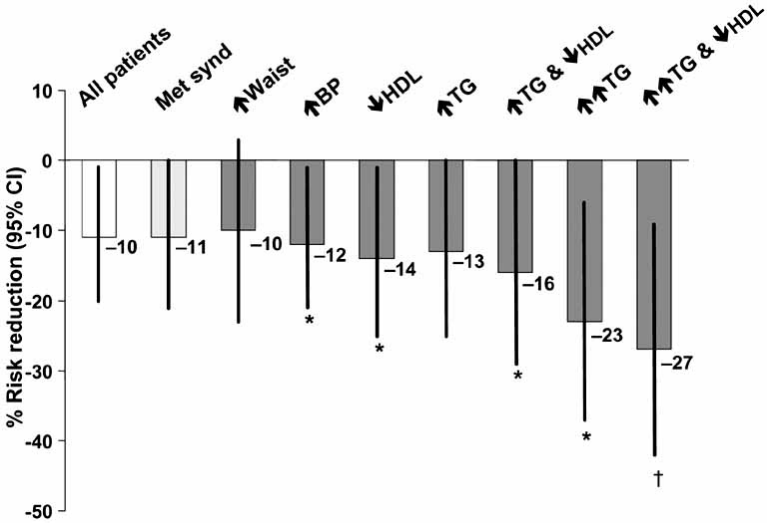
Effect of fenofibrate on cardiovascular risk in patients with features of the metabolic syndrome in the FIELD study. Reductions in risk are for fenofibrate vs. placebo. *P<0.05; †P<0.01. Data are adjusted for age, gender, prior cardiovascular disease and HbA_1c_ at baseline. Met synd: National Cholesterol Education Program/Adult Treatment Panel III metabolic syndrome. Individual criteria were as follows: **↑** waist: waist circumference >102 cm for men or >88 cm for women; **↑** BP ≥135/≥85 mmHg; **↓** HDL: HDL-C <1.03 mmol/L for men or <1.29 mmol/L for women; **↑** TG: triglycerides ≥1.7 mmol/L; **↑↑** TG: triglycerides >2.3 mmol/L. Drawn from data presented by Scott *et al.* [10].

**Table 1:** Prevalence of Features of Atherogenic Dyslipidaemia in Patients With or Without Type 2 Diabetes (T2D) in a Survey Conducted in 11 European Countries

	Men		Women	
	T2D (%)	No T2D (%)	T2D (%)	No T2D (%)
Low HDL-C	38	29	50	30
High TG	55	43	54	35
Low HDL-C and high TG	27	18	34	17
